# Supplementary material for: A scalable CRISPR/Cas9-based fluorescent reporter assay to study DNA double-strand break repair choice
Source: Nat Commun. 2020 Aug 14;11:4077. doi: 10.1038/s41467-020-17962-3 (PMC7429917; doi:10.1038/s41467-020-17962-3)
Supplement: Supplementary file 1 — Supplementary Information [file 41467_2020_17962_MOESM1_ESM.pdf]

A scalable CRISPR/Cas9-based fluorescent reporter assay to study DNA double-strand break repair choice

Paris Roidos[1#], Stephanie Sungalee[2,3#], Salvatore Benfatto[1], Özdemirhan Sercin[1], Adrian M. Stütz[2], Amir Abdollahi[4], Jan Mauer[1], Frank T. Zenke[5], Jan O. Korbel[2], Balca R. Mardin[1*]

Supplementary Figures, Figure Legends, and Tables

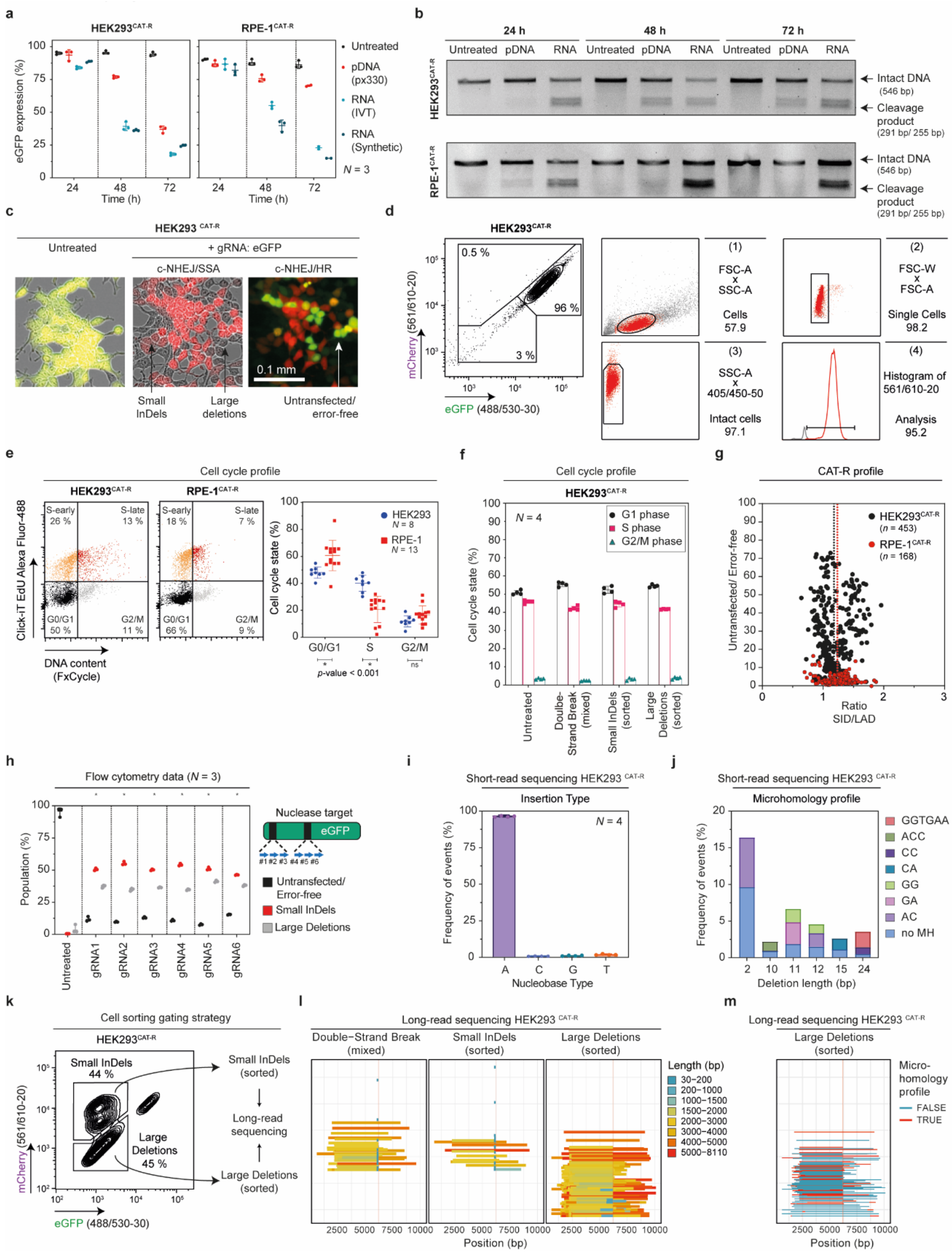

Supplementary Figure 1. CAT-R optimization.

(**a**) Scatter dot plots ($N = 3$, mean ± s.d.) show the efficiency of different gRNA formats in a time-dependent manner (24, 48, and 72 h) in HEK293^{CAT-R} and RPE-1^{CAT-R} cells. The eGFP reduction was evaluated by flow cytometry. The same gRNA sequence was used in all formats.

pDNA: px330, RNA: *in-vitro* transcribed (IVT) or synthetic (crRNA:tracrRNA) gRNA. *N* represents the number of independent experiments.

(**b**) Genomic cleavage assay of the eGFP locus targeted with the pDNA (px330), and RNA (synthetic) gRNA in HEK293$^{\text{CAT-R}}$ and RPE-1$^{\text{CAT-R}}$ cells. The cleavage efficiency of the gRNA was studied for 24 up to 72 h post-transfection with the Surveyor assay.

(**c**) Fluorescent images showing the different repair outcomes 72 h post-transfection. Only red fluorescent cells indicate repair with small InDels, whereas no fluorescence cells indicate repair by large deletions. Green & red fluorescence cells indicate the untransfected cells or cells that underwent error-free repair.

(**d**) Example of the gating strategy for flow cytometry data acquisition and analysis.

(**e**) Scatter dot plots ($N^{\text{HEK293}} = 8$; $N = 1^{\text{RPE-1}}$, mean ± s.d.) show the cell cycle profiles of untreated HEK293$^{\text{CAT-R}}$ and RPE-1$^{\text{CAT-R}}$ cells. A two-tailed t-test is used to calculate the *p-value < 0.001 between the cell lines in every cell cycle phase. *N* represents the number of independent experiments.

(**f**) Bar plots ($N = 4$, mean ± s.d.) showing the cell cycle profiles of sorted HEK293$^{\text{CAT-R}}$ cells. The cells that were transfected with gRNA:eGFP were sorted based on their CAT-R phenotype (small InDels and large deletions). Cell cycle analysis from these populations was done by incubating cells with 10 µM EdU for 1.5 h at 37°C. Cells were then processed according to the Click-iT protocol for cell cycle profile analysis. *N* represents the number of independent experiments.

(**g**) The ratio between small InDels (SID) to large deletions (LAD) upon DSB induction is unaffected by experimental conditions. Data are derived from a minimum of 56 independent experiments; *n* represents the number of all replicates.

(**h**) Flow cytometry analysis of HEK293$^{\text{CAT-R}}$ cells 72 h post-transfection with six different synthetic gRNA complexes targeting the *eGFP* coding sequence ($N = 3$, min to max, showing all points with median value). *$p \leq 0.05$ versus WT control, multiple comparison analysis testing in ANOVA followed by a Dunnett's test. All individual *p*-values are included in Source Data file 1. *N* represents the number of independent experiments.

(**i**) Box and whisker plots ($N = 4$, centerlines mark the medians, box limits indicate the 25th and 75th percentiles, and whiskers extend to min and max, showing all points) show the short-read sequencing data for small InDels representing the inserted nucleobase types at the target site. *N* represents the number of independent experiments.

(**j**) Microhomology profiles based on short-read sequencing data for small InDels.

(**k**) Fluorescence-activated cell sorting (FACS) gating strategy for downstream analysis. Samples with mCherry+/eGFP- indicated the small InDels population whereas samples with mCherry-/eGFP- to the Large Deletions population.

(**l**) Long-read sequencing data showing the profile of deletion events in different samples.

(**m**) Microhomology supporting large deletions based on long-read sequencing data for large deletions sorted sample.

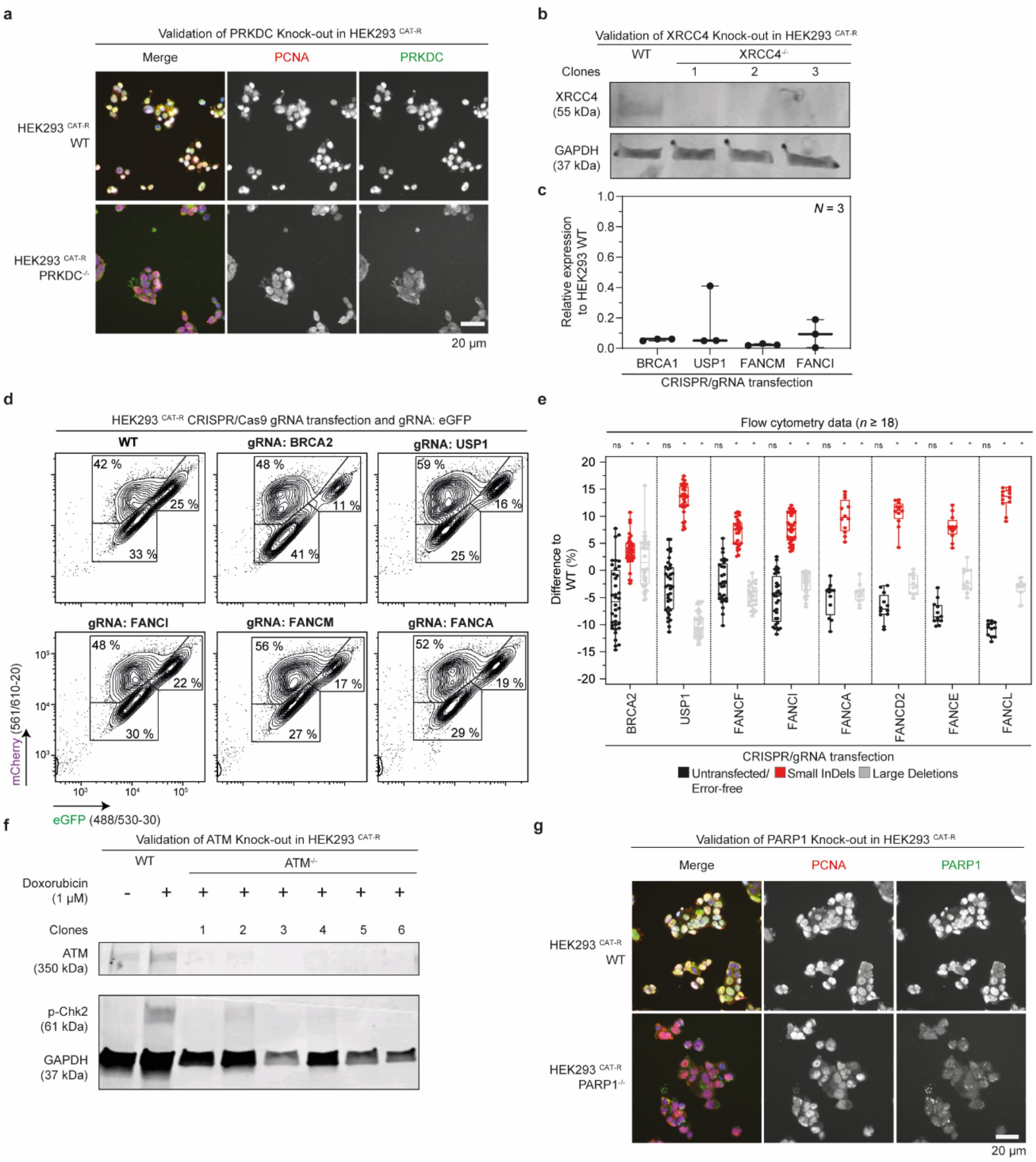

Supplementary Figure 2. CAT-R response in DDR deficient cell lines.

(**a**) Validation of the PRKDC$^{-/-}$ HEK293$^{CAT\text{-}R}$ cell line by immunofluorescence with antibodies against PCNA and PRKDC.

(**b**) Validation of the XRCC4$^{-/-}$ HEK293$^{CAT\text{-}R}$ cell line by western blot with antibodies against XRCC4 and GAPDH as control.

(**c**) Box and whisker plots (N = 3, centerlines mark the medians, box limits indicate the 25th and 75th percentiles, and whiskers extend to min and max, showing all points) of validation of reduction in mRNA levels by qPCR expression analysis in HEK293$^{CAT\text{-}R}$ 72 h post-transfection with synthetic gRNAs against BRCA1, USP1, FANCM, and FANCI gene. N represents the number of independent experiments.

(**d**) Flow cytometry analysis plot of HEK293$^{CAT\text{-}R}$ cells 72 h post-transfection with the synthetic gRNA targeting the eGFP in a mixed pool CRISPR/gRNA transfected cells. Numbers shown inside plots indicate percentages of live cells. Axes show fluorescence intensities of eGFP and mCherry proteins.

(**e**) Box and whisker plots (n^{BRAC2} = 36, n^{USP1} = 36, n^{FANCF} = 29, n^{FANCI} = 32, n^{FANCA} = 18, n^{FANCE} = 18, n^{FANCL} = 18, centerlines mark the medians, box limits indicate the 25th and 75th percentiles, and whiskers extend to min and max, showing all points) of flow cytometric analysis for HEK293$^{CAT\text{-}R}$ mixed pool CRISPR/gRNA transfected cells. Values are normalized to wildtype (WT) control, *$p \leq 0.05$ versus WT control, multiple comparison analysis testing in ANOVA followed by a Dunnett's test. All individual *p*-values are included in Source Data file 1. Data are derived from a minimum of 6 independent experiments; *n* represents the number of all replicates.

(**f**) Validation of the ATM$^{-/-}$ HEK293$^{CAT\text{-}R}$ cell line by immunoblotting with antibodies against ATM and p-CHK2 after 1.5 h incubation with 1 µM of Doxorubicin.

(**g**) Validation of the PARP1$^{-/-}$ HEK293$^{CAT\text{-}R}$ cell line by immunofluorescence with antibodies against PCNA and PARP1.

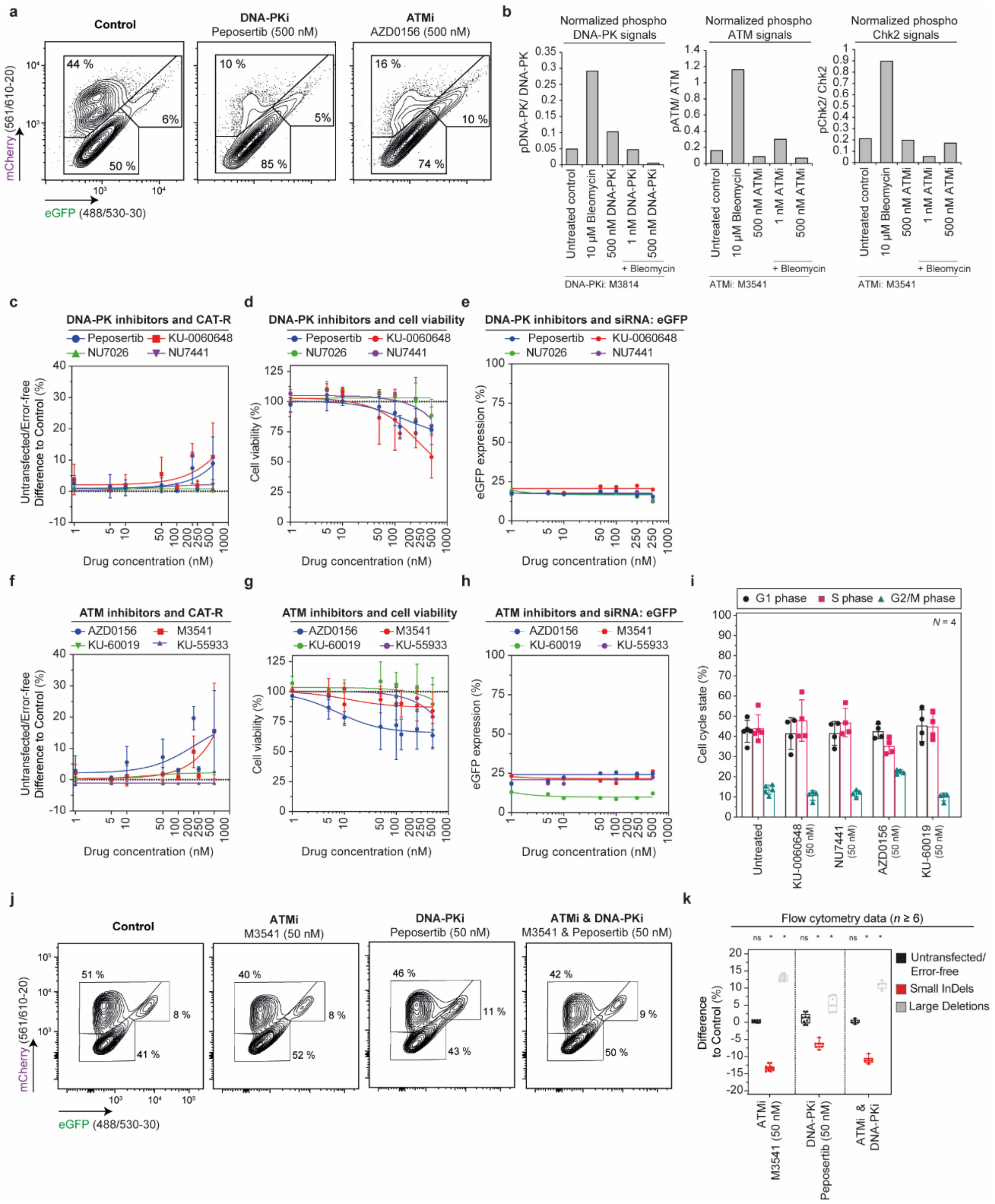

Supplementary Figure 3. Cellular responses to DNA-PK$_{cs}$ and ATM inhibition.

(**a**) Representative flow cytometry analysis plots of HEK293CAT-R cells 72 h post-transfection with the synthetic gRNA in the presence of the inhibitors: DNA-PKi (KU-0060648) and ATMi (AZD0156). Numbers shown inside plots indicate percentages of live cells. Axes show fluorescence intensities of eGFP and mCherry proteins.

(**b**) Drug target validation by immunoblotting in HEK293^{CAT-R} with antibodies against DNA-PK, ATM, CHK2, and p-CHK2 after 4 h incubation with 10 µM of Bleomycin. Barplot representing the protein levels quantification performed with the ProteinSimple Peggy Sue™ by evaluating the area under the curve for each target.

Line plots (N = 6, mean ± s.d.) of flow cytometry analysis for the $HEK293^{CAT\text{-}R}$ cells are showing the effect of (**c**) DNA-PK_{cs}, (**f**) ATM inhibitors on the frequency of the error-free population. N represents the number of independent experiments.

Cell viability assay in $HEK293^{CAT\text{-}R}$ in the presence of (**d**) DNA-PK_{cs} and (**g**) ATM inhibitors. The analysis was performed three days after treatment. Values (N = 3, mean ± s.d.) were normalized to control (DMSO). N represents the number of independent experiments.

Assessment of transfection efficiency in the presence of (**e**) DNA-PK_{cs} and (**h**) ATM inhibitors. A siRNA targeting eGFP was transfected together with the indicated inhibitors and the intensity of eGFP was measured 72 h post-transfection. Values (N = 3, mean ± s.d.) were normalized to control (DMSO). N represents the number of independent experiments.

(**i**) Bar plots (N = 4, mean ± s.d.) show the effect of DNA-PK_{cs} (KU-0060648, NU741) and ATM (AZD0156, KU-60019) inhibitors on cell cycle profile. Cells were treated for three days with 50 nM of the respective inhibitor. Cells were also incubated with 10 μM EdU for 1.5 h at 37°C. Cells were then processed according to the EdU Click-iT protocol for cell cycle analysis. N represents the number of independent experiments.

(**j**) Effect of dual ATM (M3541) and DNA-PK (Peposertib; formerly M3814) inhibition on DNA repair choice. Representative flow cytometric analysis plots of $HEK293^{CAT\text{-}R}$ cells 72 h post-transfection with the synthetic gRNA targeting the eGFP $HEK293^{CAT\text{-}R}$. Numbers shown inside plots indicate percentages of live cells. Axes report relative fluorescence intensity in arbitrary units.

(**k**) Box and whiskers plot (n^{ATMi} = 9, $n^{DNA\text{-}PKi}$ = 9, $n^{ATMi+DNA\text{-}PKi}$ = 6, centerlines mark the medians, box limits indicate the 25th and 75th percentiles, and whiskers extend to min and max, showing all points) of flow cytometry analysis for $HEK293^{CAT\text{-}R}$ cells in the presence of ATM and DNA-PK inhibitors. Values were normalized to control (DMSO), $*p \leq 0.05$ versus WT control, multiple comparison analysis testing in ANOVA followed by a Dunnett's test. All individual p-values are included in Source Data file 2. Data are derived from 3 independent experiments; n represents the number of all replicates.

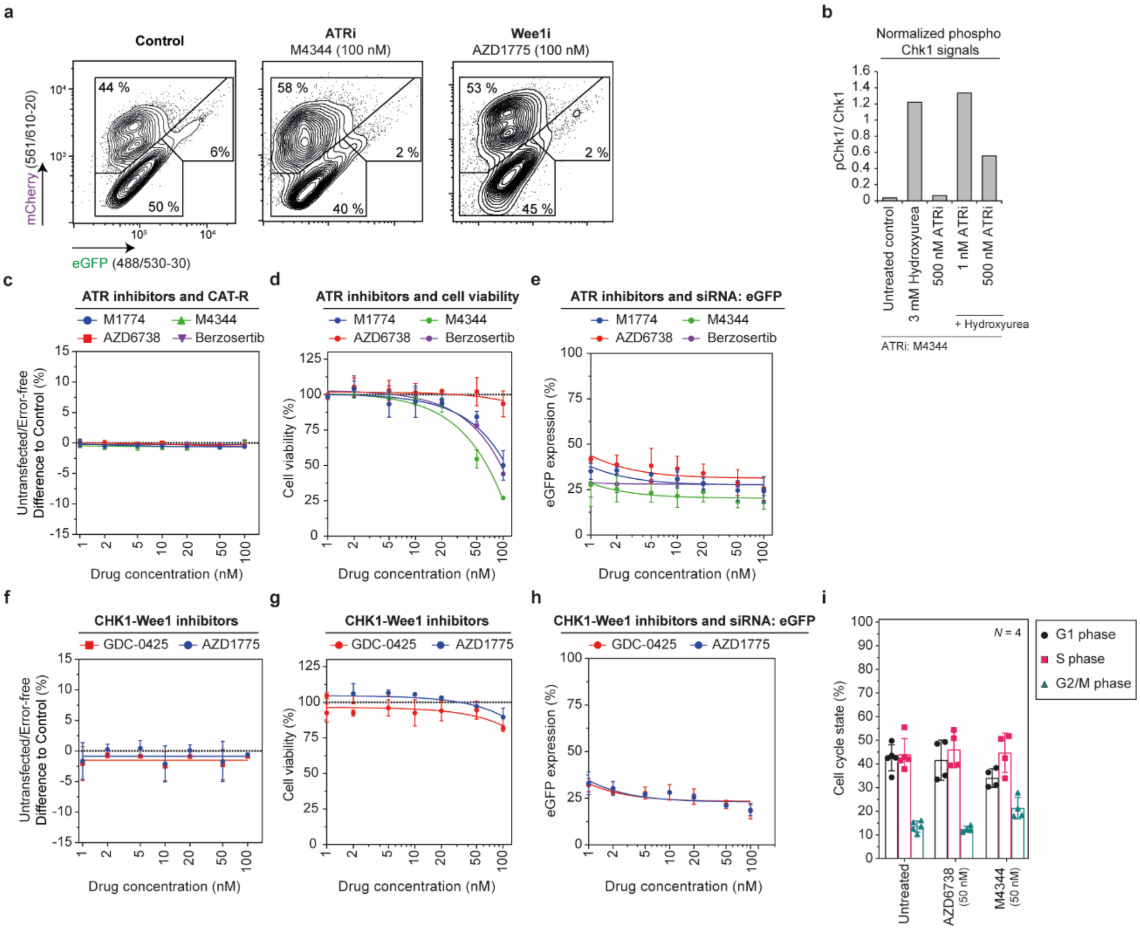

Supplementary Figure 4. Cellular responses to ATR, CHK1, and Wee1 inhibition.

(**a**) Representative flow cytometry analysis plots of HEK293$^{CAT\text{-}R}$ cells 72 h post-transfection with the synthetic gRNA in the presence of the inhibitors: ATRi (M4344) and WEE1i (AZD1775). Numbers shown inside plots indicate percentages of live cells. Axes show fluorescence intensities of eGFP and mCherry proteins.

(**b**) Drug target validation by immunoblotting in HEK293$^{CAT\text{-}R}$ with antibodies against ATR, CHK1, and p-CHK1 after 4 h incubation with 3 μM of Hydroxyurea. Protein levels quantification was performed with the ProteinSimple Peggy Sue™ by evaluating the area under the curve for each target.

Line plots (*N* = 6, mean ± s.d.) of flow cytometry analysis for HEK293$^{CAT\text{-}R}$ cells are showing the effect of (**c**) ATR, (**f**) CHK1 and WEE1 inhibitors on the frequency of the error-free population. Values were normalized to control (DMSO). *N* represents the number of independent experiments.

Cell viability assay in HEK293$^{CAT\text{-}R}$ in the presence of (**d**) ATR, (**g**) CHK1 and WEE1 inhibitors. The analysis is performed three days after treatment. Values (*N* = 3, mean ± s.d.) were normalized to control (DMSO). *N* represents the number of independent experiments.

Assessment of transfection efficiency in the presence of (**e**) ATR and (**h**) CHK1 and WEE1 inhibitors. A siRNA targeting eGFP was transfected together with the indicated inhibitors and

the intensity of eGFP was measured 72 h post-transfection. Values (*N* = 3, mean ± s.d.) were normalized to control (DMSO). *N* represents the number of independent experiments.

(**i**) Bar plots (*N* = 4, mean ± s.d.) show the effect of ATR (AZD6738 and M4344) inhibitors on the cell cycle profile. Cells were treated for three days with 50 nM of the respective inhibitor. Cells were also incubated with 10 µM EdU for 1.5 h at 37°C. Cells were then processed according to the EdU Click-iT protocol for cell cycle analysis. *N* represents the number of independent experiments.

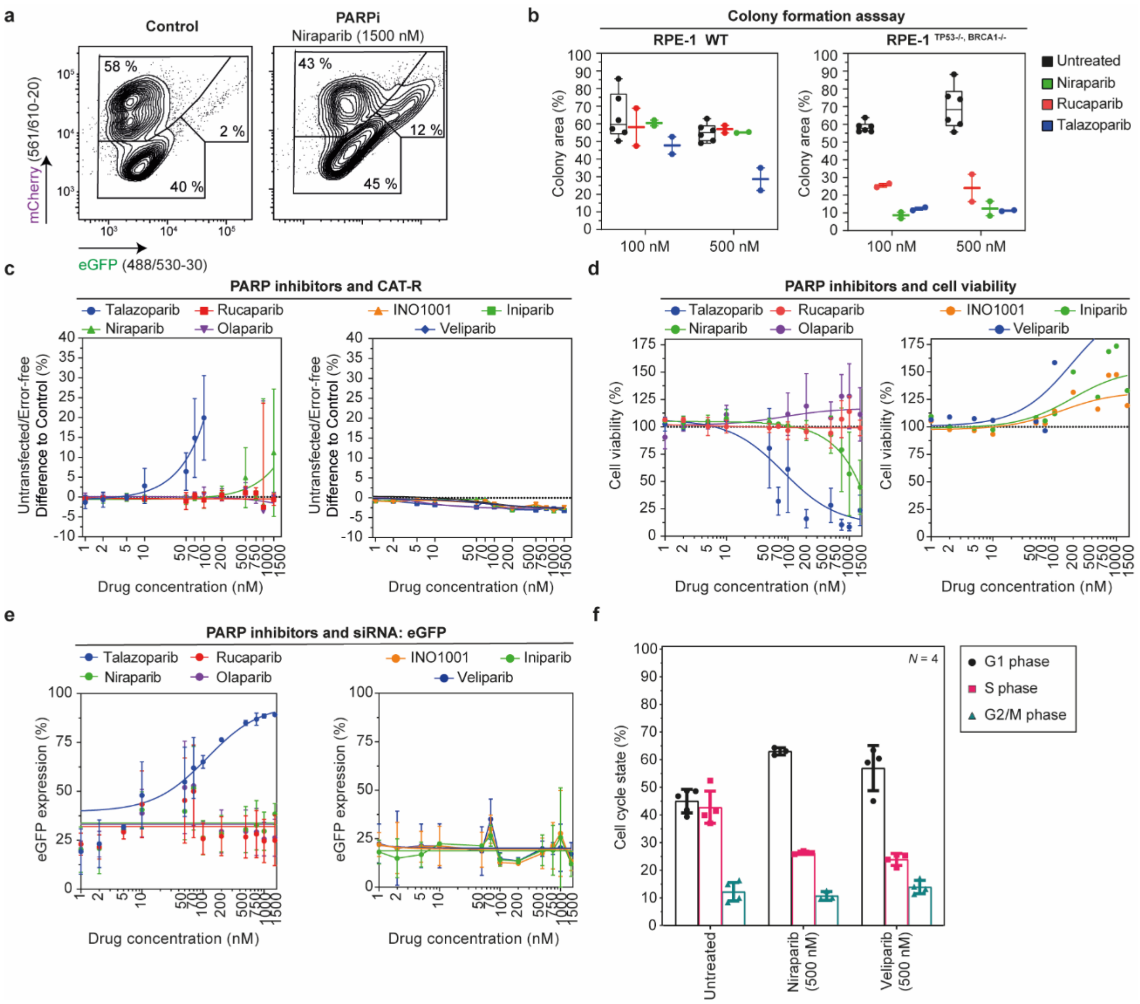

Supplementary Figure 5. Cellular responses to PARP inhibition.

(**a**) Representative flow cytometry analysis plots of HEK293$^{CAT\text{-}R}$ cells 72 h post-transfection with the synthetic gRNA in the presence of the inhibitor PARP (niraparib). Numbers shown inside plots indicate percentages of live cells. Axes show fluorescence intensities of eGFP and mCherry proteins.

(**b**) Box and whisker plots ($n^{Untreated} = 6$, $n^{Niraparib} = 2$, $n^{Rucaparib} = 2$, $n^{Talazoparib} = 2$, centerlines mark the medians, box limits indicate the 25th and 75th percentiles, and whiskers extend to min and max, showing all points) showing the dose-dependent colony formation assay of PARPi. Two concentrations of PARPi were assayed to RPE-1 and RPE-1$^{TP53,\ BRCA1}$ KO cell lines. Cells were uniformly seeded at low density (250 and 1.000 cells respectively) in individual wells of a standard 6-well plate and grown for 12 days in normal serum medium with the presence of PARPi. Colonies were visualized by crystal violet staining and quantified with ImageJ. *N* represents the number of independent experiments.

(**c**) Line plots ($N = 9$, mean ± s.d.) of flow cytometry analysis for HEK293$^{CAT\text{-}R}$ cells are showing the effect of PARP inhibitors on the frequency of the error-free population. *N* represents the number of independent experiments.

(**d**) Cell viability assay in HEK293$^{CAT\text{-}R}$ in the presence of PARP inhibitors. The analysis is performed three days after treatment. Values (*N* = 3, mean ± s.d.) were normalized to control (DMSO). *N* represents the number of independent experiments.

(**e**) Assessment of transfection efficiency in the presence of PARP inhibitors. A siRNA targeting eGFP was transfected together with the indicated inhibitors and the intensity of eGFP was measured 72 h post-transfection. Values (*N* = 3, mean ± s.d.) were normalized to control (DMSO). *N* represents the number of independent experiments.

(**f**) Bar plots (*N* = 4, mean ± s.d.) show the effect of PARP (Niraparib and Veliparib) inhibitors on the cell cycle profile. Cells were treated for three days with 500 nM of the respective inhibitor. Cells were also incubated with 10 µM EdU for 1.5 h at 37°C. Cells were then processed according to the EdU Click-iT protocol for cell cycle analysis. *N* represents the number of independent experiments.

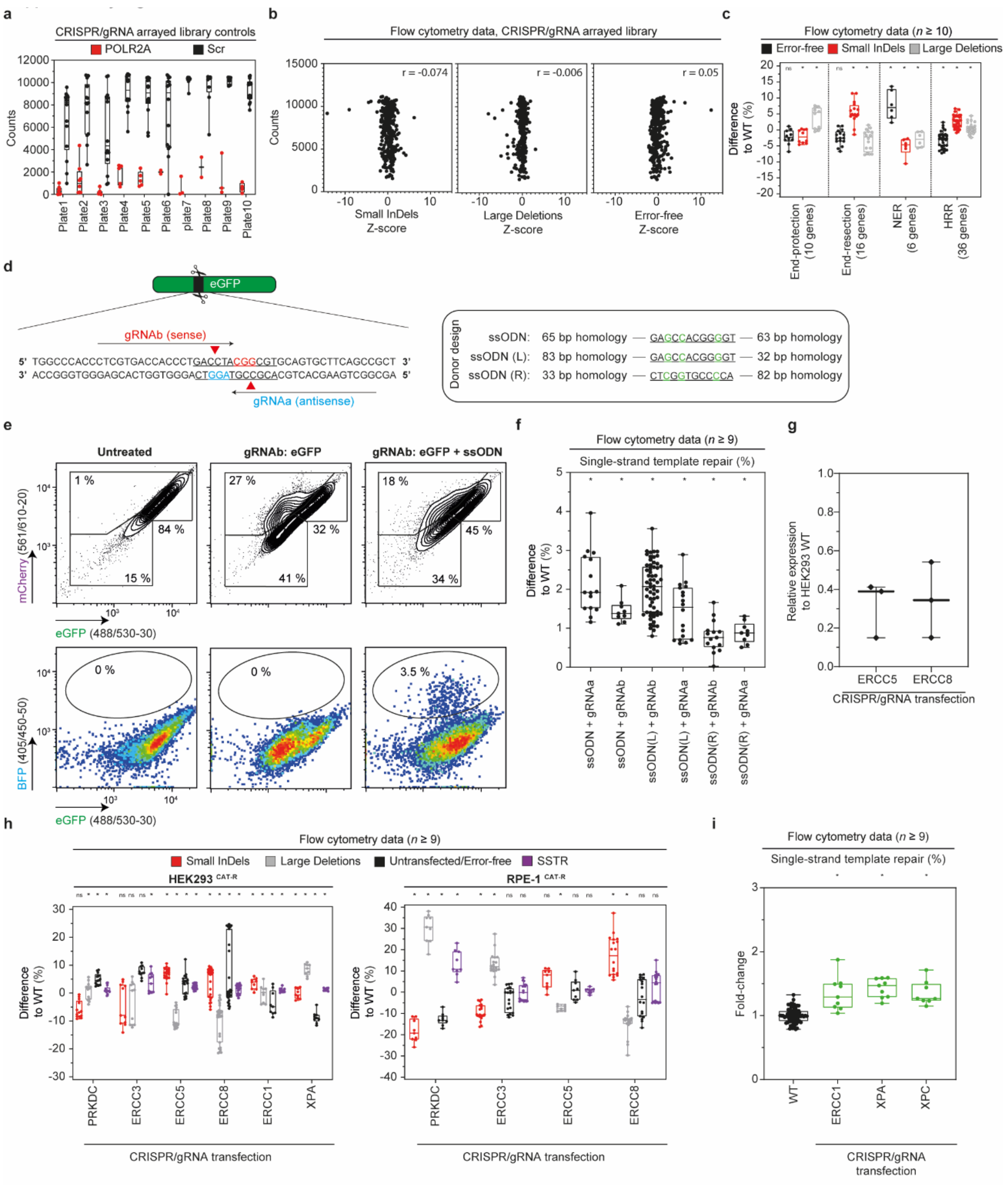

Supplementary Figure 6. CAT-R as a reporter of single-strand template repair.

(**a**) Box and whisker plots (n^{POLR2A} = 47 & n^{Scr} = 125, centerlines mark the medians, box limits indicate the 25th and 75th percentiles, and whiskers extend to min and max, showing all points) showing the transfection efficiency per 96-well plate by comparing the counts of POLR2A and Scrambled (Scr) gRNA targeted wells.

(**b**) Plots showing in the y-axis the number of acquired counts and in the x-axis the calculated z-scores values of the three populations derived from the arrayed screen. A nonparametric Spearman correlation was computed between the two data set showing that there is no correlation.

(**c**) Box and whiskers plot ($n^{\text{End-protection}} = 10$, $n^{\text{End-resection}} = 16$, $n^{\text{NER}} = 6$, $n^{\text{HRR}} = 36$, centerlines mark the medians, box limits indicate the 25th and 75th percentiles, and whiskers extend to min and max, showing all points) of flow cytometry analysis for the HEK293$^{\text{CAT-R}}$ cells. Values are normalized to wildtype (WT) control, data presented are a cluster of genes based on relevant pathways. *$p \leq 0.05$ versus WT control, multiple comparison analysis testing in ANOVA followed by a Dunnett's test. Data are derived from 2 independent experiments; *n* represents the number of all replicates.

(**d**) Schematic of Color Assay Tracing-Reporter potential outcomes after a Cas9-mediated double-stranded break in the presence of a donor template as a single-stranded oligodeoxynucleotide (ssODN). The ssODN bares two mutations that change the amino acid from Proline to Alanine so that instead of the GFP the BFP is produced indicating a knock in event via the single-strand template repair pathway. The asymmetric design of donor templates is also illustrated.

(**e**) Representative flow cytometry analysis plots of HEK293$^{\text{CAT-R}}$ cells 72 h post-transfection with the synthetic gRNA and the ssODN. Numbers shown inside plots indicate percentages of live cells. Axes report relative fluorescence intensity in arbitrary units. The conversion from GFP to BFP is quantified based on the wildtype (WT) control.

(**f**) Box and whiskers plot ($n^{\text{ssODN+gRNAa}} = 15$, $n^{\text{ssODN+gRNAb}} = 9$, $n^{\text{ssODN(L)+gRNAb}} = 60$, $n^{\text{ssODN(L)+gRNAa}} = 18$, $n^{\text{ssODN(R)+gRNAb}} = 18$, $n^{\text{ssODN(R)+gRNAa}} = 9$, , centerlines mark the medians, box limits indicate the 25th and 75th percentiles, and whiskers extend to min and max, showing all points) of flow cytometric analysis for HEK293$^{\text{CAT-R}}$ transfected with the synthetic gRNA and the different ssODN templates. *$p \leq 0.05$ versus WT control, multiple comparison analysis testing in ANOVA followed by a Dunnett's test. All individual *p*-values are included in Source Data file 3. Data are derived from 4 independent experiments; *n* represents the number of all replicates.

(**g**) Box and whisker plots ($N = 3$, , centerlines mark the medians, box limits indicate the 25th and 75th percentiles, and whiskers extend to min and max, showing all points) shows the knock-out expression levels of CRISPR/gRNA transfected cells, 72 h post-transfection with the synthetic gRNA targeting *ERCC5* and *ERCC8*, are validated by qPCR. Values are normalized to wildtype (WT) control. *N* represents the number of independent experiments.

(**h**) Box and whisker plots (For HEK293$^{\text{CAT-R}}$: $n^{\text{PRKDC}} = 13$, $n^{\text{ERCC3}} = 11$, $n^{\text{ERCC5}} = 18$, $n^{\text{ERCC8}} = 27$, $n^{\text{ERCC1}} = 9$, $n^{\text{XPA}} = 9$, For RPE-1$^{\text{CAT-R}}$: $n^{\text{PRKDC}} = 9$, $n^{\text{ERCC3}} = 19$, $n^{\text{ERCC5}} = 9$, $n^{\text{ERCC8}} = 18$, centerlines mark the medians, box limits indicate the 25th and 75th percentiles, and whiskers extend to min and max, showing all points) of flow cytometric analysis for HEK293$^{\text{CAT-R}}$ mixed pool CRISPR/gRNA transfected cells against *PRKDC*, *ERCC3*, *ERCC5*, and *ERCC8* genes. Values are normalized to wildtype (WT) control, *$p \leq 0.05$ versus WT control, multiple comparison analysis testing in ANOVA followed by a Dunnett's test. All individual *p*-values are included in Source Data file 3. Data are derived from 3 independent experiments; *n* represents the number of all replicates.

(**i**) A box and whiskers plot ($n^{\text{WT}} = 79$, $n^{\text{ERCC1}} = 9$, $n^{\text{XPA}} = 9$, $n^{\text{XPC}} = 9$, centerlines mark the medians, box limits indicate the 25th and 75th percentiles, and whiskers extend to min and max, showing all points) presenting the frequency of conversion of GFP to BFP with the use of an

asymmetric ssODN template in a mixed pool CRISPR/gRNA transfected cells. Values are represented as log2 fold change to wildtype (WT) control, *$p \leq 0.05$ versus WT control, multiple comparison analysis testing in ANOVA followed by a Dunnett's test. All individual *p*-values are included in Source Data file 3. Data are derived from 3 independent experiments; *n* represents the number of all replicates.

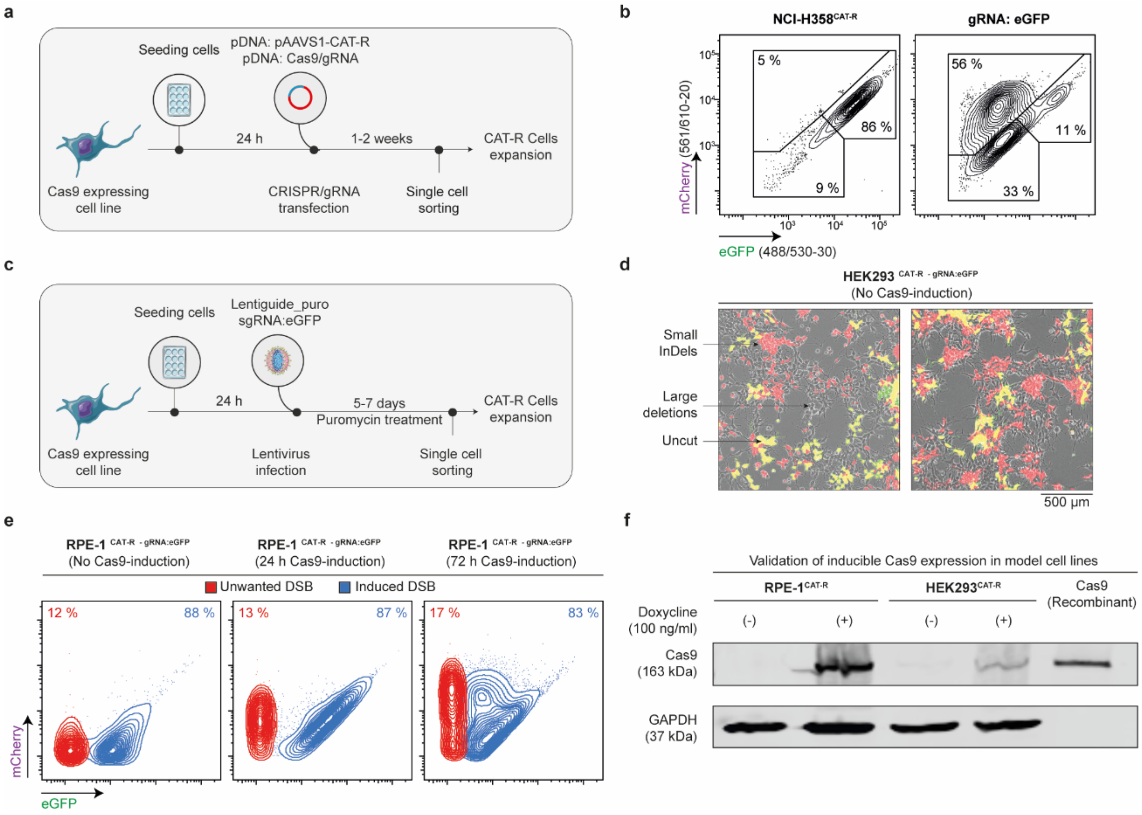

Supplementary Figure 7. Expanding the utility of CAT-R.

(**a**) The workflow of generating a CAT-R stable expressing cell line using the AAVS1 safe harbor targeting system.

(**b**) Flow cytometry plots of engineered cancer cell line NCI-H358 expressing the CAT-R system in the AAVS1 locus, 72 h post-transfection with a gRNA targeting the eGFP coding sequence. Numbers inside plots indicate percentages of live cells. Axes show fluorescence intensities of eGFP and mCherry proteins.

(**c**) The workflow of generating a gRNA: eGFP stable expressing cell using a lentivirus infection.

(**d**) Fluorescent images showing the CAT-R status in a mixed pool of uninduced HEK293$^{CAT\text{-}R\ gRNA:\ eGFP}$. Only red fluorescent cells indicate repair with small InDels, whereas no fluorescence cells indicate repair by large deletions. Green & red fluorescence cells indicate the uncut cells or cells that underwent error-free repair.

(**e**) Flow cytometry plots of engineered cell line RPE-1$^{CAT\text{-}R}$ stably expressing the gRNA: eGFP. Flow cytometry plots 24 and 72 h post Cas9-induction. Numbers inside plots indicate percentages of live cells. Axes show fluorescence intensities of eGFP and mCherry. Unwanted DSBs can be detected in the sample where Cas9 is not doxycycline induced as well as in the other samples. Please note that CAT-R system is also slightly expressed in the uninduced sample.

(**f**) Immunoblots showing inducible Cas9 expression in RPE-1 $^{CAT\text{-}R}$ and HEK293 $^{CAT\text{-}R}$ cell lines 24 h post doxycline induction.

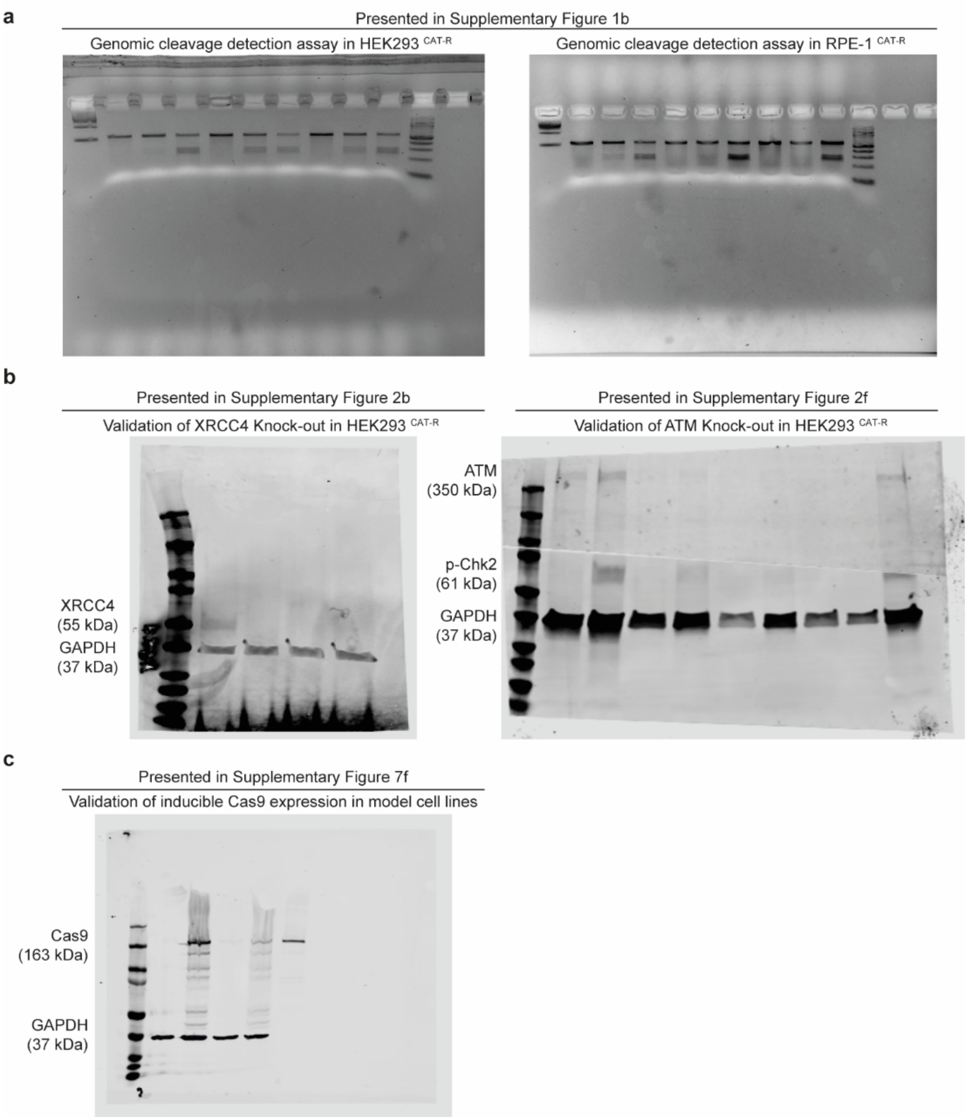

Supplementary Figure 8. Presentation of uncropped versions of gels or blots used in this study.

Supplementary Table 1. List of small pharmacological compounds.

Compound name	Target	Cancer target type/ Indications	Development stage	Company
KU-0060648	DNA-PK_{cs}	N/A	Phase I	KuDOS Ltd
Peposertib (formerly M3814)	DNA-PK_{cs}	Rectal cancer, advanced solid tumors	Phase I/II	Merck KGaA
NU7026	DNA-PK_{cs}	N/A	N/A	KuDOS Ltd
NU7441	DNA-PK_{cs}	Metastatic or locally advanced unresectable solid tumors	N/A	KuDOS Ltd
AZD0156	ATM	Advanced solid tumors	Phase I	AstraZeneca plc
KU-55933	ATM	N/A	N/A	KuDOS Ltd
KU-60019	ATM	N/A	N/A	KuDOS Ltd
M3541	ATM	Solid tumors	Phase I	Merck KGaA
AZD6738	ATR	Small Cell Lung Cancer	Phase II	AstraZeneca
M1774	ATR	N/A	N/A	Merck KGaA
M4344	ATR	Advanced solid tumors	Phase I	Merck KGaA
Berzosertib (formerly M6620)	ATR	Advanced solid tumors	Phase I	Merck KGaA
GDC-0425	CHK1	Refractory solid tumors	Phase I	Genetech Inc.
AZD1775	Wee1	Adenocarcinoma of the Pancreas	Phase II	AstraZeneca plc
Talazoparib	PARP	Locally Advanced Breast Cancer, Metastatic Breast Cancer	Phase III	Biomarin/Pfizer Inc.
Niraparib	PARP	Fallopian Tube Cancer, Ovarian Epithelial Cancer, Primary Peritoneal Cancer	Phase III	Tesaro/Merck & Co.
Rucaparib	PARP	Advanced Ovarian Cancer	Phase II	Clovis/Pfizer
Olaparib	PARP	Fallopian Tube Cancer, Metastatic Breast Cancer (MBC), Ovarian Epithelial Cancer, Refractory Advanced Ovarian Cancer	FDA approved	KuDOS/ AstraZeneca plc
Veliparib	PARP	Ovarian Cancer	Phase II	Abbott Laboratories/ AbbVie
Iniparib	PARP	Solid tumors	Phase III	Sanofi S.A.
INO-1001	PARP	Heart diseases	Phase II	Inotek/Genetech

ATM, ataxia telangiectasia mutated; ATR, AT and rad-3 related; CHK1, checkpoint kinase 1; DNA-PK_{cs}, DNA-dependent protein kinase catalytic subunit; PARP, poly adenosine diphosphate-ribose polymerase.

Supplementary Table 2. Dose-response statistics for the small pharmacological compound screens.

Compound name	Target	The goodness of Fit (least square regression model)			
		Small InDels		Large Deletions	
		R-squared	Std. Error (%)	R-squared	Std. Error (%)
KU-0060648	DNA-PK_{cs}	94.82	28.80	70.10	52.01
Peposertib (formerly M3814)	DNA-PK_{cs}	94.46	5.57	83.44	4.6
NU7026	DNA-PK_{cs}	40.64	0.25	42.37	2.92
NU7441	DNA-PK_{cs}	98.10	-	96.12	-
AZD0156	ATM	87.40	1.35	81.64	1.57
KU-55933	ATM	80.25	-	90.26	-
KU-60019	ATM	99.22	1.8	99.42	1.73
M3541	ATM	95.11	1.09	83.02	2.65
AZD6738	ATR	44.76	-	36.44	-
M1774	ATR	68.55	1.69	64.40	2.12
M4344	ATR	49.77	1.32	47.41	1.29
Berzosertib (formerly M6620)	ATR	-	3.32	12.5	1.24
GDC-0425	CHK1	71.37	3.34	51.84	-
AZD1775	Wee1	28.18	-	10.64	-
Talazoparib	PARP	65.35	0.58	43.84	-
Niraparib	PARP	43.62	1.11	2.88	1.15
Rucaparib	PARP	38.64	0.39	26.46	0.87
Olaparib	PARP	25.57	0.57	4.36	0.97
Veliparib	PARP	-	-	11.49	2.28
Iniparib	PARP	7.34	0.66	3.27	0.72
INO-1001	PARP	-	0.51	8.96	0.36

ATM, ataxia telangiectasia mutated; ATR, AT and rad-3 related; CHK1, checkpoint kinase 1; DNA-PK, DNA-dependent protein kinase catalytic subunit; PARP, poly adenosine diphosphate-ribose polymerase.

Supplementary Table 3. Oligonucleotides designs used in this study.

**gRNA sequences targeting the eGFP sequence**		
**Name**	**Orientation**	**DNA sequence (5' → 3')**
gRNA1	Sense	GGGCGAGGAGCTGTTCACCG
gRNA2	Sense	GAGCTGGACGGCGACGTAAA
gRNA3	Sense	GGCCACAAGTTCAGCGTGTC
gRNA4	Sense	GGAGCGCACCATCTTCTTCA
gRNA5	Sense	GAAGTTCGAGGGCGACACCC
gRNA6	Sense	GGTGAACCGCATCGAGCTGA
**NGS primers for short & long-read sequencing**		
**Name**	**Orientation**	**DNA sequence (5' → 3')**
CATR_short_NGS	Fwd	ACACTCTTTCCCTACACGACGCTCTTCCGATC TGGAGCGCACCATCTTCTTCAA
	Rev	CTGGAGTTCAGACGTGTGCTCTTCCGATCTGC TCAGGTAGTGGTTGTCG
CATR_long_NGS	Fwd	GCGGTCATTGACTGGAGCGA
	Rev	GCCACTTCAACATCAACGG
**PCR for surveyor assay**		
**Name**	**Orientation**	**DNA sequence (5' → 3')**
CATR_GCD	Fwd	ACGTAAACGGCCACAAGTTC
CATR_GCD	Rev	TGCTCAGGTAGTGGTTGTCG
**qPCR primers**		
**Name**	**Orientation**	**DNA sequence (5' → 3')**
BRCA1	Fwd	GCTACAGAAACCGTGCCAAA
	Rev	TATCCGCTGCTTTGTCCTCA
USP1	Fwd	CGTTTCCGGGACCAGAATCC
	Rev	CATCGCCGTCCGTTCTCTTC
FANCM	Fwd	AATCTTGGCTCTAAGTGCCAC
	Rev	TCTGCCCAATTAGCAGGTTAGTA
FANCI	Fwd	CCACCTTTGGTCTATCAGCTTC
	Rev	CAACATCCAATAGCTCGTCACC
ERCC5	Fwd	GACTTAGCGTCCAGTGACTCC
	Rev	GGCAGTTTTGATGGCTTGTCTTT
ERCC8	Fwd	ATGCTGGGGTTTTTGTCCG
	Rev	TCTCCGTGTTGACTCTGCTCT
**CAT-R: SSTR optimization**		
**Name**	**Orientation**	**DNA sequence (5' → 3')**
gRNAa	Antisense	GCTGAAGCACTGCACGCCGT
gRNAb	Sense	CTCGTGACCACCCTGACCTA
ssODN	Symmetrical	ACCCTGAAGTTCATCTGCACCACCGGCAAGC TGCCCGTGCCCTGGCCCACCCTCGTGACCAC CCTGAGCCACGGGGTGCAGTGCTTCAGCCGC TACCCCGACCACATGAAGCAGCACGACTTCT TCAAGTCCGCCATGCC

ssODN (L)	Asymmetrical	GCCACCTACGGCAAGCTGACCCTGAAGTTCATCTGCACCACCGGCAAGCTGCCCGTGCCCTGGCCCACCCTCGTGACCACCCTGAGCCACGGGGTGCAGTGCTTCAGCCGCTACCCCGACCACATGA
ssODN (R)	Asymmetrical	CTCCTGGACGTAGCCTTCGGGCATGGCGGACTTGAAGAAGTCGTGCTGCTTCATGTGGTCGGGGTAGCGGCTGAAGCACTGCACCCCGTGGCTCAGGGTGGTCACGAGGGTGGGCCAGGGCACGGGC
