# Supplementary material for: A scalable CRISPR/Cas9-based fluorescent reporter assay to study DNA double-strand break repair choice
Source: Nat Commun. 2020 Aug 14;11:4077. doi: 10.1038/s41467-020-17962-3 (PMC7429917; doi:10.1038/s41467-020-17962-3)
Supplement: Supplementary file 3 — Reporting Summary [file 41467_2020_17962_MOESM3_ESM.pdf]

nature research

Corresponding author(s): Balca R. Mardin

Last updated by author(s): Jul 10, 2020

Reporting Summary

Nature Research wishes to improve the reproducibility of the work that we publish. This form provides structure for consistency and transparency in reporting. For further information on Nature Research policies, see Authors & Referees and the Editorial Policy Checklist.

Statistics

For all statistical analyses, confirm that the following items are present in the figure legend, table legend, main text, or Methods section.

n/a	Confirmed	
☐	☒	The exact sample size (*n*) for each experimental group/condition, given as a discrete number and unit of measurement
☐	☒	A statement on whether measurements were taken from distinct samples or whether the same sample was measured repeatedly
☐	☒	The statistical test(s) used AND whether they are one- or two-sided<br>*Only common tests should be described solely by name; describe more complex techniques in the Methods section.*
☒	☐	A description of all covariates tested
☐	☒	A description of any assumptions or corrections, such as tests of normality and adjustment for multiple comparisons
☐	☒	A full description of the statistical parameters including central tendency (e.g. means) or other basic estimates (e.g. regression coefficient) AND variation (e.g. standard deviation) or associated estimates of uncertainty (e.g. confidence intervals)
☐	☒	For null hypothesis testing, the test statistic (e.g. *F*, *t*, *r*) with confidence intervals, effect sizes, degrees of freedom and *P* value noted<br>*Give P values as exact values whenever suitable.*
☒	☐	For Bayesian analysis, information on the choice of priors and Markov chain Monte Carlo settings
☒	☐	For hierarchical and complex designs, identification of the appropriate level for tests and full reporting of outcomes
☒	☐	Estimates of effect sizes (e.g. Cohen's *d*, Pearson's *r*), indicating how they were calculated

Our web collection on statistics for biologists contains articles on many of the points above.

Software and code

Policy information about availability of computer code

Data collection	LSRFortessa™ mounted on high-throughput Samples (HTS) (BD Biosciences) was used to acquire the FACS data.<br>MiSeq (Illumina) and MinION (Oxford Nanopore Technology) were used to acquire the NGS data.<br>QuantStudio3 Real Time PCR system (ThermoFisher) was used to acquire qPCR data.<br>Image Studio™ Lite software (LiCOR) was used to capture Western blot images.<br>GenCount (Oxford Optronix) was used to acquire images for the colony formation data.<br>GloMax-Multi Detection System (Promega) was used to acquire cell viability data.<br>Invitrogen™ EVOS™ Digital Color Fluorescence Microscope (FischerScientific) was used to acquire fluorescent images.
Data analysis	GraphPad Prism 8; The estimates of significance were calculated as described in the Methods section.<br>RStudio Desktop 1.3.959; Custom Rcode was used to calculate z-scores values as described in the Methods section.<br>ImageJ with 64-bit Java 1.8.0_112; “ColonyArea” plugin as described in the Methods section.<br>Excel 365; Used to analyze data and generate standard bar graphs as described in the Methods section.<br>FlowJo™ v10.6.1; Used for analyzing flow cytometry data as described in the Methods section.<br>FastQC, MultiQC, BBMap (v.38.34); Used for Data analysis of short-read sequencing.<br>guppy_basecaller (v. 3.4.5), NanoFilt (v. 2.6.0), NGMLR (v. 0.2.7), Sniffles (v. 1.0.11), pycoQC (v. 2.5.0.20), Samtools (v. 1.9); Used for Data analysis of long-read sequencing.

For manuscripts utilizing custom algorithms or software that are central to the research but not yet described in published literature, software must be made available to editors/reviewers. We strongly encourage code deposition in a community repository (e.g. GitHub). See the Nature Research guidelines for submitting code & software for further information.

Data

Policy information about availability of data

All manuscripts must include a data availability statement. This statement should provide the following information, where applicable:

- Accession codes, unique identifiers, or web links for publicly available datasets
- A list of figures that have associated raw data
- A description of any restrictions on data availability

The datasets generated during and/or analysed during the current study are available from the corresponding author upon request. Codes related to the analysis of sequencing data is available upon request. Raw amplicon sequencing data is available at the European Nucleotide Archive with the study number: PRJEB35246. The source data underlying Figure 2 and Supplementary Figure2 are provided as a Source Data file 1. The source data underlying Figure 3 is provided as a Source Data file 2. The source data underlying Figure 4 is provided as a Source Data file 3.

Field-specific reporting

Please select the one below that is the best fit for your research. If you are not sure, read the appropriate sections before making your selection.

☒ Life sciences ☐ Behavioural & social sciences ☐ Ecological, evolutionary & environmental sciences

For a reference copy of the document with all sections, see nature.com/documents/nr-reporting-summary-flat.pdf

Life sciences study design

All studies must disclose on these points even when the disclosure is negative.

Sample size	No sample-size calculation was performed. Specifically for examining the robustness of the method, 151 biological experiments were performed with HEK293 and 56 biological experiments with RPE-1 cell lines. A minimum of 3 biological replicates in all experiments was conducted. In each biological replicate, we included 3 technical replicates.
Data exclusions	For FACS data, samples with counts < 1.500 were excluded from subsequent analyses as described in Material and Methods section.
Replication	We define biological replicates as completely independent experiments carried out on different days with a different batch of materials. In each biological replicate, whenever technically possible we included 3 replicates (e.g. 3 wells of a 96-well plate). We performed a minimum of 3 independent experiments to ensure reproducibility.
Randomization	All samples were analyzed equally with no sub-sampling thus, there was no requirement for randomization.
Blinding	Not relevant to this study. Quantifications were performed using computational pipeline applied equally to all conditions and replicates for a given data set.

Reporting for specific materials, systems and methods

We require information from authors about some types of materials, experimental systems and methods used in many studies. Here, indicate whether each material, system or method listed is relevant to your study. If you are not sure if a list item applies to your research, read the appropriate section before selecting a response.

Materials & experimental systems

n/a	Involved in the study	
☐	☒	Antibodies
☐	☒	Eukaryotic cell lines
☒	☐	Palaeontology
☒	☐	Animals and other organisms
☒	☐	Human research participants
☒	☐	Clinical data

Methods

n/a	Involved in the study	
☒	☐	ChIP-seq
☐	☒	Flow cytometry
☒	☐	MRI-based neuroimaging

Antibodies

Antibodies used	Mouse anti-Cas9 (IF, 1:1000, Cell Signaling, cat:14697s)<br>Rabbit anti-ATM (IF, 1:1000, Cell Signaling, cat:2873s)<br>Rabbit anti-pChk2 (IF, 1:1000, Cell Signaling, cat:2197s)<br>Mouse anti-XRCC4 (IF, 1:500, Santa Cruz, cat:sc-271087)<br>Rabbit anti-DNAPKcs (IF, 1:1000, Abcam, cat:ab70230)<br>Rabbit anti-PARP1 (IF, 1:1000, Cell Signaling, cat:9542)

	Rabbit anti-GAPDH (IF, 1:10.000, Cell Signaling, cat:5174s)<br>Goat anti-mouse or anti-rabbit IR680 or IR800 (IB, 1:10.000, Licor,cat:296-32213 and 296-32211).
Validation	All antibodies were validated by the respective manufacturers. In addition, antibody specificity was evaluated using the proper negative controls. In addition, bands were identified at expected molecular weights for protein products.

Eukaryotic cell lines

Policy information about cell lines

Cell line source(s)	HEK293 (Flp-In™-293 Cell Line, ThermoFisher Scientific),<br>RPE-1 (a kind gift from Jonathon Pines; originally derived from ATCC as hTERT RPE-1 (ATCC® CRL-4000™)),<br>NCI-H358 (ATCC)
Authentication	None of the cell lines used were authenticated.
Mycoplasma contamination	All cell lines were regularly tested negative for mycoplasma contamination.
Commonly misidentified lines<br>(See ICLAC register)	None of the used cell lines used were listed in ICLAC database.

Flow Cytometry

Plots

Confirm that:

☒ The axis labels state the marker and fluorochrome used (e.g. CD4-FITC).

☒ The axis scales are clearly visible. Include numbers along axes only for bottom left plot of group (a 'group' is an analysis of identical markers).

☒ All plots are contour plots with outliers or pseudocolor plots.

☒ A numerical value for number of cells or percentage (with statistics) is provided.

Methodology

Sample preparation	Cell medium was removed, and cells were washed with PBS. Then they were detached with TrypLE™ dissociating reagent and after 10' of incubation, they were re-suspended in equal amounts of DMEM (no phenol red).
Instrument	FACS LSRFortessa™ (BD Biosciences)
Software	FlowJo_v10.6.1
Cell population abundance	More than 98% determined by post sort analysis.
Gating strategy	Cell populations were gated on a forward- (FSC)/side- scatter (SSC) plot. Cells were further gated on forward-area (FSC-A)/ forward-height scatter (FSC-H) plot to determine single cells. Single cells were further gated on side-area scatter (SSC-A)/ (405-450/50A) to determine living cells based on DAPI staining. Live cells were further gated to determine eGFP (488-530/30-A)/ mCherry (561-610/20-A) cell populations and evaluate in a ratiometric way the fluorescent variations.

☒ Tick this box to confirm that a figure exemplifying the gating strategy is provided in the Supplementary Information.
